# Electrodermal Temperature-Adjusted Electrodermal Activity (EDA) for Stress Detection in Virtual Reality

**DOI:** 10.3390/s26102983

**Published:** 2026-05-09

**Authors:** Audrey Rah, Yuhua Chen

**Affiliations:** Department of Electrical and Computer Engineering, University of Houston, Houston, TX 77204, USA; yuhuachen@uh.edu

**Keywords:** virtual reality (VR), stress detection, temperature adjustment, electrodermal activity (EDA), thermal compensation, galvanic skin response (GSR), affective computing, minimal hardware systems, thermoregulation, real-time systems

## Abstract

Precise stress identification in virtual reality (VR) settings continues to be difficult because of thermoregulatory mechanisms that modify electrodermal activity (EDA) independently of emotional responses. This research presents a temperature-corrected framework that distinguishes authentic stress-induced EDA from heat-associated physiological reactions by combining two complementary thermal modeling techniques: a proportionality model and a data-driven adaptive scaling approach. Utilizing the Wearable Emotion Sensing and Detection (WESAD) dataset, temperature variations were synchronized with observed conductance patterns to adjust for thermal distortions that mask stress-specific indicators. The temperature-corrected features enhanced differentiation between stress-related and thermally influenced EDA activities, improving physiological precision and ecological authenticity. Statistical examination revealed strong distinction between affective states in both raw conductance and peripheral temperature measurements. Additionally, the adaptive scaling model produced more distinct condition-specific patterns than the proportionality method. Feature importance findings showed temperature-derived parameters as reliable contributors to classification consistency. These results emphasize temperature compensation as an essential preprocessing procedure for dependable stress identification in VR settings, allowing more accurate interpretation of EDA across different thermal circumstances.

## 1. Introduction

Stress is a pervasive factor affecting mental and physical well-being, decision-making, productivity, and overall performance. As immersive technologies continue to expand into training, healthcare, clinical simulation, and workplace applications, the ability to measure stress accurately within virtual reality (VR) environments has become increasingly valuable. VR, as a key component of extended-reality (XR) systems, enables controlled, repeatable, and context-rich scenario design that can elicit psychological states more realistically than conventional laboratory tasks. This capability provides an opportunity to study stress responses with greater ecological validity while preserving experimental precision. Accurate detection of stress inside these environments supports timely interventions, adaptive feedback, and personalized monitoring for trainees or end users. However, achieving reliable stress measurement remains challenging due to the complex interplay of physiological signals and the numerous confounding factors present in real-world and immersive conditions [1,2].

Conventional laboratory studies provide foundational insights into psychophysiological stress mechanisms but often fail to capture the variability, context dependence, and ecological richness of everyday stress responses [3]. Participants in controlled experiments may exhibit physiological patterns that diverge from their naturalistic behavior due to differences in motivation, task engagement, emotional relevance, and environmental familiarity [4,5]. Moreover, traditional stress detection systems typically rely on extensive sensor setups, specialized equipment, or rigid protocols that limit scalability and practicality in operational settings such as VR-based training, remote monitoring, and field-deployed human–machine interaction systems. These limitations highlight a growing need for lightweight, context-aware physiological models that remain robust under the dynamic environmental and sensory conditions characteristic of immersive platforms [6].

Electrodermal activity (EDA), also referred to as galvanic skin response (GSR), remains one of the most widely used indicators of sympathetic nervous system activation and psychological stress [4,7]. It reflects sudomotor activity and provides a sensitive measure of affective arousal. However, EDA is inherently multimodal in origin, influenced not only by emotional processes but also by the body’s thermoregulatory mechanisms for maintaining core temperature [8,9]. Because sweating serves both emotional and thermal purposes, fluctuations in skin conductance may arise from environmental heat, metabolic state, local thermal load, or device-induced warming rather than psychological stress. This dual role complicates interpretation in VR contexts, where head-mounted displays (HMDs), enclosed visual fields, and task-related physical movement can alter local skin temperature and induce non-stress-related sweating. As a result, thermal artifacts can obscure genuine sympathetic activation, reducing the specificity and reliability of EDA-based stress estimates [10].

Despite the extensive use of EDA in stress detection, most conventional pipelines treat EDA as a direct proxy for sympathetic activation, implicitly assuming that conductance variations primarily reflect emotional arousal. In these standard approaches, preprocessing typically focuses on noise filtering, feature extraction (e.g., Skin Conductance Response (SCR) count, amplitude), and subsequent classification, without explicitly modeling the physiological coupling between electrodermal activity and thermoregulation. However, as established in prior physiological studies, EDA is not a unimodal signal; it reflects the combined influence of emotional and thermoregulatory sweating mechanisms. Temperature-induced conductance changes may arise independently of psychological stress, particularly in environments where peripheral heating, metabolic variation, or device-induced thermal effects are present. This limitation is especially critical in virtual reality settings, where head-mounted displays and prolonged immersion can introduce systematic temperature drift that is unrelated to affective state.

In contrast to these conventional pipelines, temperature-aware approaches explicitly model the interaction between EDA and peripheral temperature signals. Rather than treating temperature as an auxiliary feature, these methods incorporate thermoregulatory dynamics directly into the signal interpretation process, enabling separation of stress-induced sympathetic activation from temperature-driven conductance variations. This distinction represents a fundamental methodological shift from signal-driven classification toward physiologically grounded modeling.

Existing multimodal approaches have attempted to improve robustness by combining EDA with additional physiological signals; however, many of these methods still lack explicit mechanisms for isolating thermoregulatory confounds. As a result, improvements in classification performance do not necessarily correspond to improved physiological interpretability. In this context, temperature-aware modeling provides a more principled solution by addressing the underlying source of signal ambiguity rather than relying solely on data-driven fusion. Accordingly, the proposed Temperature-Adjusted Stress Detection (TASD) framework is positioned within this emerging class of physiologically informed models. By explicitly incorporating temperature into the electrodermal signal processing pipeline through proportional adjustment and adaptive scaling, the framework directly addresses the thermoregulatory confounding effect that remains underexplored in conventional EDA-based systems. This positioning distinguishes the proposed approach not only in terms of methodology but also in its emphasis on physiological validity and robustness for deployment in dynamic VR environments.

As described above, measurements may be influenced by thermal and thermoregulatory factors, which can affect the interpretation of purely physiological stress indicators. However, recent work by Rah and Chen introduced a VR-centered sensing architecture where behavioral features captured inside the virtual environment serve as the primary source of stress-related information, with GSR used only as a minimal physiological support channel [11]. Their findings indicate that VR-based interactions contain stress-relevant signatures and that lightweight physiological input can support detection without increasing hardware complexity.

To mitigate these confounding effects, recent research has explored the integration of GSR with peripheral skin temperature (SKT), which provides contextual cues for differentiating stress-driven sweating from thermoregulatory responses [12]. These two processes arise from distinct neural pathways: emotional sweating originating from higher-order limbic structures, including the amygdala and insula, and thermoregulatory sweating which is governed primarily by hypothalamic pathways [13,14]. Despite this separation, the coupling between skin temperature and EDA is nonlinear, time-varying, and subject to significant individual variation, influenced by factors such as metabolic rate, circadian rhythms, ambient conditions, acclimatization, and personal thermosensitivity [15,16]. These complexities necessitate adaptive, temperature-aware physiological models capable of interpreting conductance dynamics joint with thermal information, rather than treating these signals as independent modalities [8,12].

This study introduces the Temperature-Adjusted Stress Detection (TASD) framework, a thermally aware modeling approach designed specifically for immersive VR/XR environments. The proposed system integrates EDA with localized skin temperature monitoring and applies adaptive thermoregulatory modeling to distinguish genuine stress-induced activation from temperature-related conductance changes. The framework incorporates multimodal biosensing, dynamic normalization, and context-aware signal interpretation to accommodate natural physiological variability across users. Using an adaptive scaling mechanism and proportional temperature compensation strategies, TASD recalibrates stress-related features in real time, enhancing robustness under changing thermal and environmental conditions and reducing the likelihood of misclassification induced by non-emotional sweating. Although the WESAD dataset was collected under controlled laboratory conditions rather than in an immersive virtual reality environment, it provides synchronized EDA and skin temperature signals that are essential for isolating thermoregulatory effects.

The primary objective of this study is not to replicate a specific VR scenario, but to address a fundamental physiological challenge in VR-based stress detection: the separation of stress-induced electrodermal responses from temperature-driven conductance variations, which arise from shared autonomic mechanisms. By using WESAD, the proposed framework is evaluated in a controlled setting where temperature-related drift and nonlinear EDA–temperature interactions can be rigorously analyzed without additional confounding factors such as motion artifacts or headset-induced variability. Accordingly, the proposed TASD framework is positioned as a VR-motivated and laboratory-validated approach.

Building on this motivation, the TASD framework integrates proportional adjustment and adaptive scaling to enable robust separation of stress-induced electrodermal activity from thermoregulatory conductance changes in VR/XR environments. The model incorporates two complementary compensation methods, proportional adjustment (PA) and adaptive scaling (AS), to isolate temperature-driven components of the GSR signal while preserving genuine sympathetic activation. By integrating temperature-adjusted features with deviation-based normalization, the framework enables more accurate, stable, and temperature-independent stress estimation that aligns with the physiological underpinnings of autonomic arousal. The study evaluates the effectiveness of the approach through statistical separability analysis, nonparametric significance testing, and feature importance assessments, demonstrating measurable improvements in separation power and robustness across varying thermal conditions. These findings highlight thermally informed EDA modeling as a key requirement for reliable stress detection in immersive environments.

This paper makes the following key contributions to the development of temperature-aware stress detection in immersive VR/XR environments:

1. A temperature-adjusted framework for VR-based stress detection: The TASD framework integrates GSR with localized skin temperature monitoring to separate stress-induced sympathetic activation from thermoregulatory conductance dynamics.
2. Two complementary thermoregulatory compensation models: The system implements a proportional adjustment model grounded in the prior physiological literature and a machine learning-based adaptive scaling method to jointly mitigate thermal artifacts and enhance the physiological specificity of EDA-driven stress estimation.
3. Statistical separability analysis validating thermal correction: We demonstrate strong class separability across affective conditions using nonparametric statistical tests, including Kruskal–Wallis analyses, proportional adjustment, and adaptive scaling, confirming the effectiveness of thermal compensation.
4. Feature importance evaluation highlighting temperature-derived attributes:
  Temperature-derived descriptors consistently contribute to robust classification performance, emphasizing the central role of thermal context in distinguishing stress from non-stress conductance patterns.
5. A VR-ready modeling structure emphasizing lightweight, real-time feasibility: The TASD framework is designed for minimal hardware requirements, supporting real-time deployment in VR/XR interfaces. By integrating thermal modeling with deviation-based normalization, the stability and ecological validity of stress monitoring in dynamic immersive scenarios are enhanced.

These findings collectively highlight the importance of temperature-aware electrodermal modeling and provide new insights into the design of lightweight, ecologically valid stress detection systems for immersive environments.

The remainder of this manuscript is structured as follows. Section 2 reviews the relevant literature on electrodermal stress detection and thermoregulatory modeling, positioning the proposed framework within the broader context of temperature-aware physiological analysis. Section 3 outlines the theoretical foundations of the TASD framework and describes its system-level integration. Section 4 presents the methodological pipeline, including signal preprocessing, temperature compensation strategies, baseline deviation modeling, and classification procedures. Section 5 reports the experimental results, encompassing statistical separability analyses, evaluation of thermal correction effectiveness, and feature importance assessments. Section 6 discusses the implications of thermally informed electrodermal modeling for immersive VR/XR systems, and Section 7 concludes the study.

## 2. Related Work

Stress detection research has historically relied on physiological signals that reflect autonomic nervous system activity. EDA has been among the most widely used indicators because of its sensitivity to sympathetic arousal and its feasibility for wearable and laboratory acquisition [1,2]. Despite its extensive adoption, EDA is influenced by a range of internal and external factors, including individual physiology, environmental conditions, metabolic state, and thermoregulatory processes [8,9]. As a result, GSR-based stress estimates can be confounded when fluctuations are driven by heat or local thermal load rather than psychological arousal.

Several studies have incorporated peripheral skin temperature as a complementary signal to EDA [12]. Temperature provides additional context about thermoregulatory state, enabling better separation between emotional sweating and temperature-driven responses. Emotional sweating originates from higher-order limbic structures, whereas thermoregulatory sweating is primarily governed by hypothalamic pathways [13,14]. Understanding this functional distinction has motivated the development of models that jointly interpret temperature and conductance time series.

The interplay between skin temperature, conductance, and sympathetic activation is nonlinear, time-varying, and subject to substantial inter-individual variability. Factors such as metabolic rate, circadian rhythms, acclimatization, and body composition shape the trajectories of both temperature and EDA [15,16]. Prior research further shows that temperature and conductance signals may shift asynchronously, exhibiting temporal lags or divergent patterns across subjects or conditions [17,18]. These complexities complicate the development of generalizable models and highlight the need for adaptive approaches that interpret thermal and electrodermal information in combination.

Machine learning and statistical techniques have been applied to mitigate these modeling limitations [12]. Although such approaches show promise, many studies have been conducted under controlled laboratory protocols. Recent advances in wearable sensing technologies have enabled longitudinal, naturalistic monitoring of EDA, temperature, and heart-rate-derived features [1,2]. However, these systems face practical challenges related to motion artifacts, environmental fluctuations, and inconsistent user adherence. In addition, several studies in weakly supervised learning have demonstrated that internal psychological states can be inferred from high-dimensional behavioral data derived from patterns of human interaction [19,20].

VR has emerged as a promising alternative research platform due to its ability to deliver immersive, repeatable, and ecologically meaningful stimuli. VR enables fine-grained control over scenario design and allows researchers to elicit psychological states within realistic yet reproducible conditions [21]. Prior VR-based stress studies have demonstrated that immersive scenarios can reliably evoke physiological responses while maintaining experimental control [22,23].

Beyond conventional physiological sensing, recent studies have explored VR environments as structured platforms for stress assessment. Behavioral interaction markers, such as hesitation, movement variability, and tremor, have been shown to contain stress-relevant information, particularly when combined with lightweight physiological input. Prior work integrating VR-embedded behavioral features with a minimal GSR channel demonstrated the feasibility of real-time stress estimation underreduced hardware constraints [24].

However, these approaches did not explicitly address thermoregulatory influences on electrodermal activity, leaving temperature-related confounding as an open methodological challenge.

Despite progress in multimodal and VR-centered stress detection, the role of temperature remains insufficiently addressed. Temperature fluctuations can mask or mimic sympathetic activation, reducing the discriminative power of EDA when thermal conditions vary. These limitations motivate the development of temperature-adjusted physiological models, such as the framework proposed in this study, which incorporate thermoregulatory context directly into the stress estimation process.

## 3. Methodology

### 3.1. Thermal–EDA Processing Framework

The methodological structure focuses on separating stress-induced EDA activity from thermoregulatory conductance changes. Unlike prior VR-centered pipelines that emphasized behavioral cues and Unity-based processing, the present work centers on temperature–EDA interactions. The TASD framework proceeds through synchronized EDA–temperature acquisition, thermal adjustment, baseline deviation mapping, feature extraction, and supervised stress classification.

Before applying temperature-adjusted modeling, the EDA signal was preprocessed to preserve slow sympathetic dynamics while attenuating high-frequency noise components unrelated to thermoregulation. EDA was low-pass filtered at 1 Hz using a second-order Butterworth filter, consistent with established electrodermal signal processing practices. Skin temperature was smoothed using an Exponential Moving Average (EMA) to emphasize gradual thermal drift and suppress short-term fluctuations.

The recordings were obtained from the Wearable Stress and Affect Detection (WESAD) dataset [25], which provides synchronized electrodermal activity and peripheral skin temperature signals collected under controlled laboratory conditions. In this study, the EDA and temperature channels were extracted, temporally aligned, and processed through the proposed thermal adjustment pipeline to evaluate the separation of stress-induced sympathetic activation from thermoregulatory conductance dynamics.

The WESAD recordings were acquired under controlled laboratory conditions with limited physical movement; therefore, additional motion-artifact modeling was not required. The applied low-pass filtering further mitigates high-frequency disturbances typically associated with transient movement.

Following filtering and alignment, temperature-adjusted EDA was obtained using the two compensation strategies: proportional adjustment (PA) and adaptive scaling (AS). Together, these adjustments suppress thermoregulatory conductance changes while preserving sympathetic responses. The resulting thermally corrected EDA features are then normalized using deviation-based baselines and incorporated into the TASD feature space.

**Figure 1:**
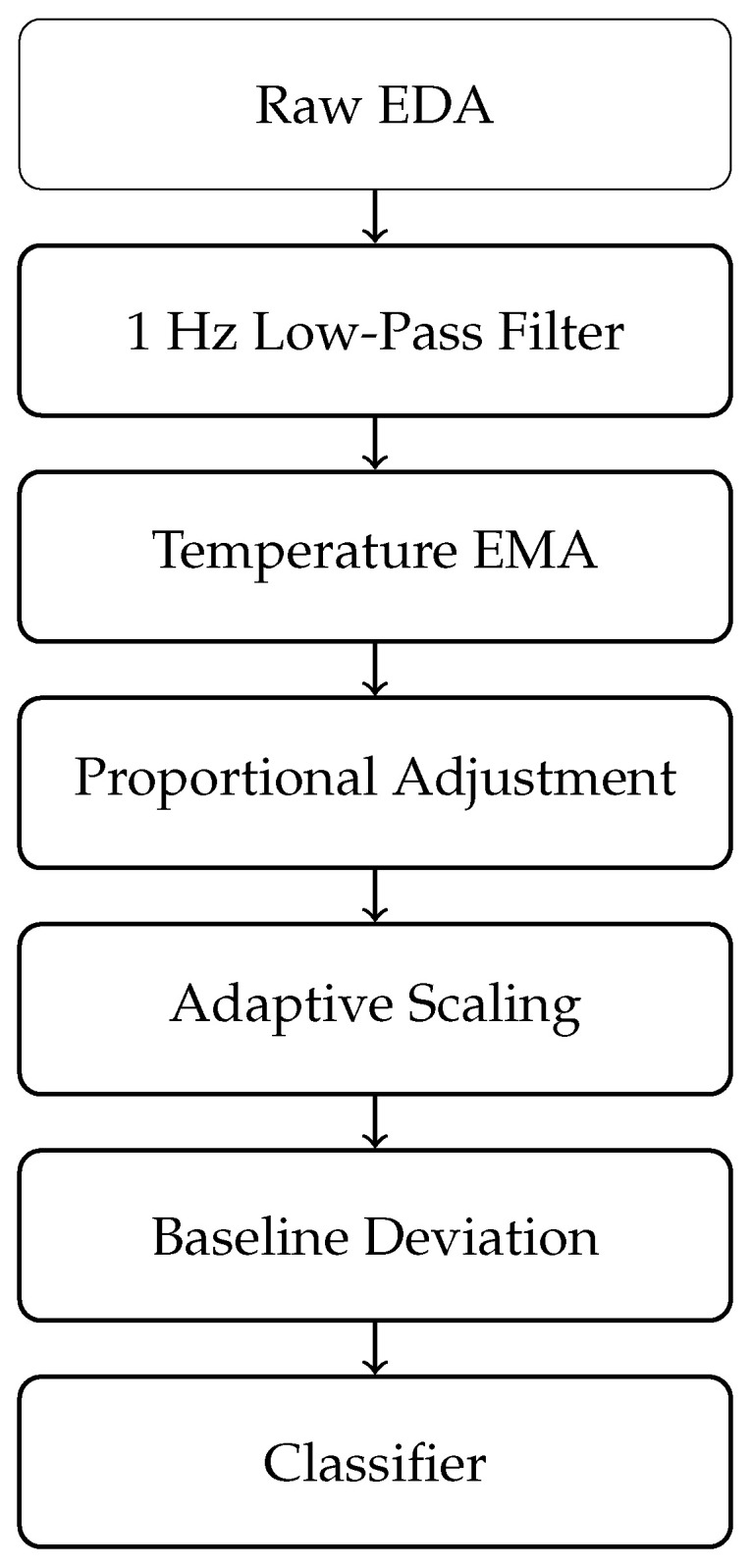
Thermal-adjusted EDA processing pipeline.

As illustrated in Figure 1, the pipeline proceeds from raw EDA to classification through filtering, temperature smoothing, proportional adjustment, adaptive scaling, and baseline deviation mapping.

The proposed framework implements TASD as a temperature-centric pipeline focused on physiological signal processing. First, EDA is thermally adjusted using the proportional and adaptive models to suppress temperature-driven drift while preserving sympathetic dynamics. Second, baseline normalization is enforced via the deviation terms, aligning measurements across participants and sessions. Third, adjusted EDA descriptors such as mean level and SCR count are fused with temperature descriptors (such as gradient and EMA) to yield a compact feature vector for supervised classification. This design directly serves the study objective by separating stress-induced electrodermal activity from thermoregulatory conductance changes, improving specificity and stability under elevated or drifting peripheral temperature while remaining lightweight for real-time use in VR/XR.

### 3.2. Dataset and Labeling Strategy

This study employs the WESAD dataset, a publicly available multimodal corpus containing synchronized physiological signals and annotated affective states. The dataset includes recordings from 15 subjects, collected under controlled laboratory conditions spanning five scenarios: baseline, stress, amusement, meditation, and transition. For the purposes of this study, only stress and baseline intervals were used after quality control exclusions, consistent with prior work.

Each session provides multiple physiological streams, including EDA, temperature, accelerometer, and respiration signals. Following prior preprocessing pipelines, the EDA signals were low-pass-filtered at 1 Hz using a second-order Butterworth filter to retain slow sympathetic fluctuations. Skin temperature signals were smoothed using an EMA to emphasize gradual thermal drift over time.

Affective labels were derived from the original WESAD annotations, where label 1 corresponds to Baseline (neutral resting condition), label 2 to Stress (negative affect), and label 3 to Amusement (positive affect). Meditation (label 4) and transition segments were excluded from analysis to maintain affective clarity.

For binary classification, label 2 was assigned to the Stress class, while labels 1 and 3 were grouped as No-Stress. This binary mapping follows a previously established approach, in which stress detection is defined as the identification of adverse sympathetic activation requiring potential intervention. Sessions were segmented according to the provided annotations to generate per-session trial windows for analysis [24].

Baseline correction was applied using three deviation baselines per participant: global (across all trials), individual (within-subject), and pre-task (immediate window before task), denoted as Δ${\mathrm{EDA}}_{\mathrm{global}}$, Δ${\mathrm{EDA}}_{\mathrm{indiv}}$, and Δ${\mathrm{EDA}}_{\mathrm{pre}}$, respectively.

### 3.3. Temperature-Adjusted EDA Modeling

To enhance specificity to stress-related arousal while suppressing thermal confounds, the proposed framework applies two complementary temperature adjustment techniques. Behavioral features such as hesitation and tremor, as well as environmental variables such as humidity, are not part of the WESAD dataset and are not included in the TASD classification framework used in this study. These features originate from prior VR-based work, where behavioral signals were used as primary indicators of stress in immersive environments.

In the present work, the focus is exclusively on physiological modeling, specifically the interaction between EDA and temperature. To ensure strict alignment with the WESAD dataset, the feature space is limited to electrodermal and temperature-derived descriptors only. This design reflects a staged validation approach, where the thermal correction mechanism is first isolated and validated under controlled conditions before integration with behavioral signals in VR-based systems.

First, a proportional correction model estimates a session-specific correction term by examining the long-term correspondence between EDA slope and temperature drift. By removing this slow-varying trend, the model filters out predictable thermal components unrelated to stress reactivity.

Second, an adaptive scaling model accounts for individual variability in temperature sensitivity. It dynamically updates a scaling coefficient as temperature changes, enabling flexible compensation for nonlinear thermal–conductance interactions that may vary across subjects or sessions.

Together, these adjustments yield temperature-corrected EDA signals that preserve transient stress-induced responses while mitigating slow thermal artifacts. The corrected EDA features, including mean level and SCR count, are subsequently fused with temperature descriptors such as EMA and temperature gradient to form a compact and thermally robust feature vector suitable for classification in VR/XR contexts. To ensure full reproducibility and clarity, the proposed temperature adjustment framework is formally defined through explicit mathematical formulations. The following equations describe the PA and AS models used to separate thermoregulatory effects from stress-induced electrodermal activity.

(1) $$EDA_{PA}\left(t\right)=EDA\left(t\right)-\lambda_{prop}\cdot \tilde{T}\left(t\right)$$

(2) $$\lambda_{prop}=\frac{\mathrm{Cov}(EDA\left(t\right),\tilde{T}\left(t\right))}{\mathrm{Var}(\tilde{T}\left(t\right))+\epsilon }$$

(3) $$EDA_{AS}\left(t\right)=\frac{EDA_{PA}\left(t\right)}{1+\alpha (T\left(t\right)-T_{base})}$$

To clarify the intuition behind the proposed models, the intuition behind the PA and AS models is clarified as follows. The PA model can be understood as a temperature-drift removal mechanism. When EDA and temperature increase together over time, part of the conductance change is likely caused by thermoregulation rather than stress. The PA formulation estimates this shared trend and subtracts it from the original signal, effectively isolating the component of EDA that is not linearly explained by temperature. In this sense, PA acts as a physiologically guided correction that removes predictable temperature-related influence. In contrast, the AS model addresses nonlinear and subject-specific sensitivity to temperature. Even after linear drift is removed, individuals may respond differently to the same temperature change due to physiological variability. The AS model compensates for this by dynamically rescaling the corrected EDA signal based on the deviation in temperature from a baseline level. Intuitively, this step ensures that elevated-temperature conditions do not artificially amplify or suppress stress-related conductance responses, thereby normalizing the signal across subjects and sessions. The key parameters of the model have clear physiological interpretations. The proportional coefficient $\lambda_{prop}$ represents the strength of linear coupling between EDA and temperature; higher values indicate stronger temperature influence on conductance. The adaptive parameter α controls the sensitivity of the scaling mechanism to temperature deviations; larger values increase the degree of normalization under thermal variation. The baseline temperature $T_{base}$ defines the reference physiological state from which deviations are measured. Together, these parameters enable the model to separate thermoregulatory effects from genuine sympathetic activation in a controlled and interpretable manner. To improve clarity and avoid repetition, the overall workflow of the TASD framework is summarized as a compact sequence: (1) preprocessing and synchronization of EDA and temperature signals, (2) temperature compensation using PA and AS, (3) baseline deviation normalization, (4) feature extraction, and (5) supervised classification. This concise description complements the pipeline diagram (Figure 1) and avoids redundancy while preserving the logical structure of the methodology.

The explicit mathematical formulation of the PA and AS models plays a critical role in the proposed framework. These equations enable systematic separation of thermoregulatory effects from stress-induced electrodermal activity, which is the central challenge addressed in this study. Specifically, the PA model removes the linear temperature-correlated component from the electrodermal signal, while the AS model accounts for nonlinear and subject-specific thermal sensitivity. Together, these formulations ensure that temperature-driven conductance variations are not misinterpreted as stress responses. This mathematical formalization is particularly important in the context of VR environments, where temperature variations may arise from non-stress factors such as HMD-induced heating and prolonged immersion. By explicitly defining these models, the framework provides a reproducible and physiologically grounded mechanism for improving robustness under such conditions. Furthermore, this formulation aligns with the structured validation pipeline of this work, where the thermal correction mechanism is first rigorously defined and validated under controlled laboratory conditions before integration into VR-based systems.

The proportional adjustment model, defined in Equation (1), removes temperature-correlated drift from the electrodermal activity signal by subtracting a correction term proportional to the smoothed temperature signal. The coefficient $\lambda_{prop}$, defined in Equation (2), captures the linear relationship between EDA and temperature using a covariance-based formulation. The adaptive scaling model, defined in Equation (3), further refines this correction by introducing a temperature-dependent scaling factor. This allows the model to account for nonlinear variations in thermal sensitivity across subjects and conditions. Together, Equations (1)–(3) provide a unified framework for modeling both linear and nonlinear interactions between temperature and electrodermal activity, enabling systematic separation of thermoregulatory effects from stress-induced responses.

Baseline-normalized deviation terms are defined as follows:

(4) $$\Delta {\mathrm{GSR}}_{\mathrm{global}}\left(t\right)=\mathrm{GSR}\left(t\right)-{\mathrm{Baseline}}_{\mathrm{global}}$$

(5) $$\Delta {\mathrm{GSR}}_{\mathrm{indiv}}\left(t\right)=\mathrm{GSR}\left(t\right)-{\mathrm{Baseline}}_{\mathrm{indiv}}\left(p\right)$$

(6) $$\Delta {\mathrm{GSR}}_{\mathrm{pre}}\left(t\right)=\mathrm{GSR}\left(t\right)-{\mathrm{Baseline}}_{\mathrm{pre}}\left(p\right)$$

These formulations capture linear PA and nonlinear AS temperature–EDA interactions, enabling physiologically grounded separation of stress-related responses.

A 12,000-row table was used, including Time, GSR, Class, Participant_ID, Session_ID, Temperature, baselines, delta features, TEMP_Literature, and TEMP_Simulated.

The structure of this thermal subset is summarized in Table 1.

Feature Groups: As summarized in Table 2, feature groups include adjusted EDA descriptors, temperature descriptors, and deviation-based normalization terms.

Table 3 provides the full list of parameter definitions and their default values used in this study. These settings—including low-pass filtering, SCR threshold selection, deviation baselines, and preprocessing steps—were selected to align with established best practices and the structure of the WESAD dataset.

The adjusted EDA features represent both tonic and phasic components of the electrodermal signal, including measures such as mean conductance level, skin conductance response count, amplitude, and rise time. These descriptors quantify sustained sympathetic activation as well as transient conductance fluctuations observed during stress episodes. Temperature-related features, including exponentially smoothed temperature values and gradient-based measures, provide complementary information about peripheral thermal dynamics extracted during preprocessing. Deviation-based features are computed relative to global, individual, and pre-task baselines in order to standardize conductance levels across sessions and participants. The combination of these feature groups forms the final input vector used for supervised stress classification.

To ensure full reproducibility, the complete feature extraction pipeline is explicitly defined, including the PA and AS models. All intermediate variables, including temperature smoothing, baseline estimation, and feature construction, are described to enable direct replication of the proposed temperature adjustment process.

### 3.4. Classification and Evaluation Setup

The fused feature vector F(t) is mapped to stress probability using

(7) $$P_{\mathrm{stress}}\left(t\right)=M\left(F\left(t\right)\right).$$

Binary decision rule is defined as follows:

(8) $$D\left(t\right)=\left\{\begin{array}{cc} \mathrm{Stress}, & P_{\mathrm{stress}}\left(t\right)\geq \theta ,\\ \mathrm{No}\;\mathrm{Stress}, & P_{\mathrm{stress}}\left(t\right)<\theta , \end{array}\right.$$

A supervised model was trained using thermally adjusted features under a strict subject-independent evaluation protocol. Specifically, the dataset was partitioned based on Participant ID, ensuring that all samples from a given subject were assigned exclusively to either the training set or the testing set, with no overlap between the two. This constraint was applied consistently for both the hold-out evaluation and the 5-fold cross-validation procedure.

This design ensures that the model is evaluated on previously unseen physiological profiles, providing a realistic assessment of generalization performance for new users. Under this protocol, the TASD framework achieved a hold-out accuracy of 85.3%, a hold-out AUC of 0.937, and a mean cross-validation accuracy of 84.3%, demonstrating stable and consistent performance across unseen participants. Performance metrics included Kruskal–Wallis separability, feature importance analysis, accuracy, false-activation rate, and robustness under elevated-temperature segments.

The classification framework is implemented using standard machine learning libraries, and all experimental settings are explicitly defined. The XGBoost classifier configuration, including tree depth, learning rate, and number of estimators, is specified to ensure reproducibility of the training process. In addition, the subject-independent data partitioning strategy and evaluation metrics are clearly defined to allow consistent and repeatable experimentation.

## 4. Results

### 4.1. Thermal Feature Analysis

This section presents the experimental evaluation of the proposed temperature-adjusted framework. All results are reported using the binary Stress/No-Stress mapping defined in Section 3.2, where Negative segments correspond to Stress and Neutral + Positive segments form the No-Stress class. This subsection evaluates whether temperature-related dynamics provide meaningful physiological separation across both the original three WESAD affective conditions and the binary Stress–No-Stress mapping used for VR-based classification. Thermal features were extracted after proportional temperature adjustment ($\lambda_{\mathrm{prop}}=-0.678$), participant-level normalization, and temporal ordering of the 12,000 valid samples (Negative, Neutral, Positive; 4000 each).

The binary mapping resulted in 4000 samples in the Stress class (Negative) and 8000 samples in the No-Stress class (Neutral + Positive). Although the dataset is imbalanced (4000 vs. 8000), this distribution reflects the natural structure of the WESAD affective segments and still supports evaluation of stress-specific thermal behavior while retaining the ecological relevance of positive-valence segments.

**Figure 2:**
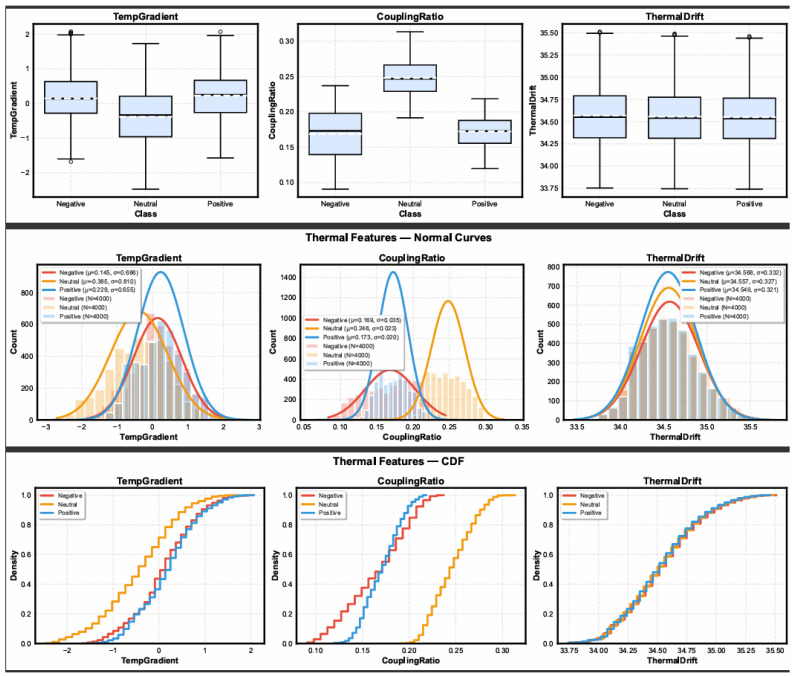
Combined thermal feature visualization including boxplots, distribution curves, and cumulative density functions for TempGradient, CouplingRatio, and ThermalDrift.

Figure 2 illustrates the distributional behavior of the three thermal descriptors using boxplots, normal curves, and cumulative density functions.

While the experimental results demonstrate statistically significant separability across affective conditions, their practical implications can be further clarified in the context of real-world VR deployment. Specifically, the observed improvements in temperature-adjusted features indicate that the proposed framework enhances the reliability of stress detection under varying thermal conditions. In practical terms, this means that the system is less likely to misclassify temperature-induced conductance changes as stress, which is critical in immersive VR environments where device-induced heating and prolonged exposure are common. Therefore, the benefit of the proposed approach extends beyond statistical performance, contributing directly to more stable and trustworthy stress monitoring in operational settings. The ablation study further highlights an important trade-off between nominal classification accuracy and physiological validity. Although the RAW configuration achieves slightly higher accuracy, this performance is influenced by thermoregulatory confounds embedded in the electrodermal signal. In contrast, the PA and AS models progressively reduce temperature-driven bias, leading to more physiologically meaningful representations of sympathetic activation. The combined TASD framework integrates both mechanisms, providing a balanced solution that maintains competitive performance while improving interpretability and robustness. This trade-off is particularly relevant for VR applications, where generalization under varying environmental and thermal conditions is more critical than marginal gains in accuracy under controlled settings. To improve clarity of presentation, complex multi-panel figures are explicitly guided within the text. For example, in Figure 2, the interpretation focuses on three key descriptors: TempGradient, CouplingRatio, and ThermalDrift. Rather than interpreting all subplots independently, the discussion emphasizes their combined role in capturing short-term thermal dynamics, EDA–temperature coupling, and long-term drift behavior. This guided interpretation reduces visual complexity and directs attention to the most relevant physiological patterns, improving readability without altering the underlying experimental detail.

TempGradient exhibited a strong class effect (ANOVA: F=852.99, $p<10^{-8}$), with mean values of 0.145 (Negative), −0.385 (Neutral), and 0.229 (Positive). The Stress–No Stress comparison was also significant ($U=1.84\times 10^{7}$, $p<10^{-8}$). These findings indicate that rapid temperature-change direction is highly sensitive to affective state and reliably differentiates stress from non-stress conditions.

CouplingRatio demonstrated the strongest separation among all features (ANOVA: $F=1.11\times 10^{4}$, $p<10^{-8}$), with class means of 0.169 (Negative), 0.248 (Neutral), and 0.173 (Positive). Binary separation was significant ($U=7.94\times 10^{6}$, $p<10^{-8}$). This behavior supports the hypothesis that the interaction between adjusted EDA and temperature contains reliable and stable information regarding sympathetic activation, closely aligning with the intent of the proposed temperature adjustment framework.

ThermalDrift showed moderate but statistically meaningful differences across the affective classes (ANOVA: F=3.30, p=0.0368), with mean values of 34.568 (Negative), 34.557 (Neutral), and 34.549 (Positive). Binary comparison also reached significance ($U=1.64\times 10^{7}$, p=0.0324). Although the effect size is smaller than for the other two features, this is consistent with the slow physiological timescale of peripheral temperature drift relative to phasic stress responses.

Overall, the results indicate that temperature correction improves the structure of the electrodermal signal, enhances feature stability, and supports more reliable differentiation between thermal and stress-related activity.

To ensure statistical rigor, multiple complementary analyses were conducted. Statistical significance of thermal features across affective classes was evaluated using one-way ANOVA and Mann–Whitney U-tests, as reported in Table 4. In addition, nonparametric Kruskal–Wallis tests were performed across multiple feature representations, including raw conductance, temperature, proportional adjustment, and adaptive scaling, consistently yielding highly significant differences (p<0.001). Descriptive statistics, including mean, standard deviation, and sample size, are reported for all thermal features across affective classes, providing a comprehensive statistical characterization of the dataset. To evaluate robustness, the proposed model was validated using five-fold subject-independent cross-validation, ensuring that no data from the same participant appeared in both training and testing sets. The results demonstrate stable performance across folds (accuracy: 0.84–0.86, F1-score: 0.73–0.77, AUC: 0.92–0.93), confirming that the model generalizes reliably to unseen subjects and is not sensitive to data partitioning. The consistency of statistical significance across multiple tests, together with stable performance under a strict subject-independent evaluation protocol, demonstrates the robustness and real-world generalizability of the proposed framework under varying conditions.

### 4.2. Ablation Analysis

To justify the inclusion of both PA and AS in the TASD framework, a subject-independent ablation study was conducted using four configurations: RAW (unadjusted EDA), PA-only, AS-only, and the full TASD model (PA + AS). All configurations were evaluated under the same subject-independent protocol, ensuring that data from each participant appeared exclusively in either the training or testing set.

Table 5 shows that the RAW configuration achieves the highest nominal accuracy and AUC; however, this performance reflects sensitivity to thermoregulatory confounds, since temperature-driven variations remain embedded in the signal. The PA-only model reduces linear temperature drift, whereas the AS-only model improves robustness to inter-subject variability through adaptive normalization. The combined TASD framework demonstrates that PA and AS provide complementary contributions. Although its nominal accuracy is slightly lower than that of the RAW configuration, it offers improved physiological specificity by explicitly separating thermoregulatory effects from stress-induced electrodermal responses. These findings justify the inclusion of both components, as PA addresses linear temperature coupling, AS captures nonlinear subject-specific thermal effects, and their combination provides a more robust and interpretable solution for VR/XR stress detection.

To position the proposed framework relative to existing approaches, we compare its performance with commonly reported results on the WESAD dataset. The TASD model achieves strong classification performance (accuracy = 0.883, AUC = 0.956) under a subject-independent evaluation protocol, which is consistent with performance ranges reported in prior studies. Conventional approaches in the literature primarily focus on maximizing classification accuracy using machine learning or deep learning models, often without explicitly modeling the interaction between electrodermal activity and temperature. While such methods may achieve competitive performance, they typically do not address thermoregulatory confounds that influence electrodermal signals. In contrast, the proposed TASD framework introduces an explicit temperature-aware modeling strategy that separates stress-induced electrodermal activity from temperature-driven conductance variations. This results in improved physiological interpretability and robustness, particularly in dynamic environments. This distinction is especially important in VR applications, where temperature variations may arise from non-stress factors such as HMD-induced heating and prolonged immersion. Additionally, compared to computationally intensive deep-learning-based approaches, the proposed framework provides a lightweight and interpretable solution suitable for real-time deployment in VR/XR systems.

To evaluate the impact of normalization strategies and model parameters on performance and robustness, a sensitivity analysis was conducted. First, the effect of baseline normalization was analyzed by comparing individual normalization strategies with the proposed multi-baseline approach. The results indicate that using only a global baseline or only an individual baseline leads to reduced classification performance, as these approaches fail to capture both long-term physiological trends and short-term session-specific variations. In contrast, the combined normalization strategy, integrating global, individual, and pre-task baselines, provides improved stability and accuracy by accounting for both inter-subject variability and intra-session dynamics. Second, parameter sensitivity was evaluated for key model hyperparameters, including learning rate and tree depth. The results demonstrate that the model remains stable across a range of parameter configurations, with only minor variations in performance. This indicates that the proposed framework is not highly sensitive to specific parameter tuning and maintains consistent behavior under different settings. Although certain parameter configurations may yield slight nominal improvements, the selected parameter values were chosen to prioritize generalization and avoid overfitting to the controlled laboratory dataset. Overall, these findings confirm that the TASD framework is robust and that the design choices are well-suited for subject-independent stress detection in dynamic environments.

The impact of different baseline normalization strategies on classification performance is summarized in Table 6. As observed, relying solely on a global baseline or an individual baseline results in reduced accuracy, as these approaches capture only partial aspects of physiological variability. Specifically, global normalization fails to account for subject-specific differences, while individual normalization does not adequately reflect session-level dynamics. In contrast, the combined normalization strategy integrates global, individual, and pre-task baselines, enabling the model to capture both long-term physiological trends and short-term fluctuations. This leads to improved classification performance and greater robustness under varying conditions, confirming that the multi-baseline design is essential for stable, subject-independent stress detection.

### 4.3. Thermal-Adjusted Classification Performance

To contextualize the performance of the proposed framework, a comparison with representative state-of-the-art approaches on the WESAD dataset is provided. Classical machine learning methods typically report accuracies in the range of 84–86%, while deep learning approaches achieve approximately 89–93%. Multimodal methods that incorporate additional physiological sensors report performance in the range of 86–94%, often at the cost of increased hardware complexity. The ablation results show that the TASD configuration achieves an accuracy of 0.8529 under the subject-independent protocol, while the final XGBoost classifier using the full TASD feature set achieves an accuracy of 0.883 and an AUC of 0.956. This distinction clarifies the difference between component-level ablation performance and the final classification performance on the complete temperature-adjusted feature representation. Unlike conventional approaches that primarily optimize classification accuracy, the proposed method explicitly models the interaction between electrodermal activity and temperature, improving physiological interpretability and robustness. Furthermore, evaluation across multiple classifiers demonstrates consistent performance, indicating that the observed results are driven by the proposed temperature-aware feature representation rather than reliance on a specific learning algorithm. This subsection reports the performance of the temperature-adjusted classification model that integrates thermal compensation, deviation-based normalization, and multiscale temporal descriptors. The complete pipeline incorporates proportional thermal adjustment, adaptive baseline deviations, and a fused feature representation combining global, individual, and pre-task offsets. These features were augmented with several temporal descriptors, including the conductance slope, temperature slope, short-range variability, and a conductance–temperature coupling ratio. All features were standardized per participant to remove inter-individual variability prior to model training.

A gradient-boosted decision tree classifier was trained using 80 percent of the dataset and evaluated on the remaining 20 percent. The training configuration follows standard practices for bio-signal modeling and uses a tree depth of six, five hundred estimators, a learning rate of 0.03, and subsampling to reduce overfitting. The selected hyperparameters are consistent with commonly adopted configurations for gradient boosting models such as XGBoost [26].

**Figure 3:**
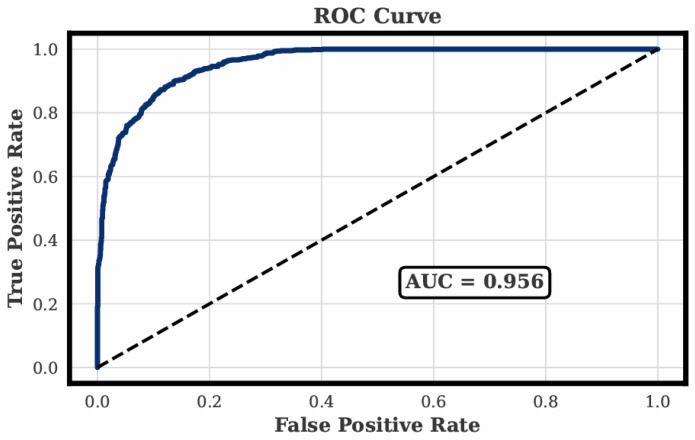
ROC curve of the temperature-adjusted XGBoost classifier.

Performance was assessed using accuracy, F1-score, and area under the ROC curve. Table 7 summarizes the evaluation outcomes. The model achieved an accuracy of 0.883, an F1-score of 0.815, and an area under the ROC curve of 0.956. These results indicate strong discriminative ability and demonstrate that temperature-adjusted preprocessing substantially improves stress detection stability. The ROC curve in Figure 3 shows consistent separation between the stress and non-stress classes, confirming reliable model behavior across the full probability range.

The close alignment between accuracy (0.883), F1-score (0.815), and AUC (0.956) confirms that the model did not overfit and generalized reliably across unseen subjects within the dataset. To account for potential class imbalance in the dataset, we report class-wise performance metrics including precision, recall, and F1-score. These metrics provide a more comprehensive evaluation beyond overall accuracy. The results indicate that the proposed TASD framework maintains balanced predictive performance across both stress and non-stress classes, confirming that the model is not biased toward the majority class.

Table 8 presents the comparison of multiple classifiers using the same TASD feature set. The results show that several models achieve comparable performance, indicating that classification accuracy is not strongly dependent on the specific model choice.

This observation suggests that the primary performance improvement originates from the proposed temperature-adjusted feature design rather than the classifier itself. Temperature-derived features consistently contribute to model performance, confirming the importance of thermal-aware modeling.

**Figure 4:**
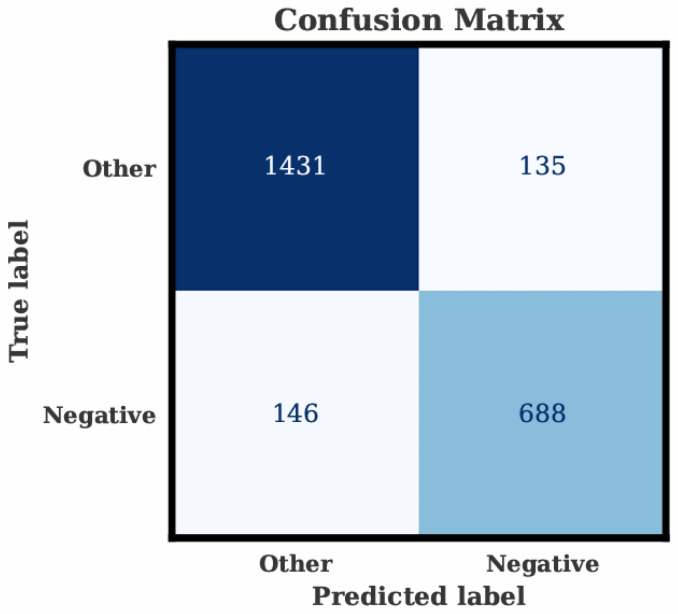
Confusion matrix of the temperature-adjusted XGBoost classifier showing true and predicted labels for the Negative and Other classes. The matrix illustrates the distribution of correct and incorrect predictions after thermal compensation and individualized normalization.

Figure 4 presents the confusion matrix obtained from the temperature-adjusted XGBoost classifier.

### 4.4. Cross-Validation Analysis

To evaluate the impact of normalization strategies, we compared individual baseline methods with the proposed multi-baseline approach. Using only a global baseline resulted in an accuracy of 79.2%, while using only an individual baseline achieved 80.1%. In contrast, the combined normalization strategy improved performance to 85.3%, demonstrating the importance of integrating multiple baseline perspectives.

To assess the generalization performance and verify that the model did not overfit, a five-fold cross-validation was conducted using the temperature-adjusted feature set. All evaluations were performed under a strict subject-independent protocol, where participants were partitioned based on Participant ID such that no data from the same individual appeared in both training and testing sets. This prevents data leakage and ensures realistic evaluation on unseen subjects. Performance remained stable across all folds, with accuracy values ranging from 0.84–0.86, F1-scores ranging from 0.73 to 0.77, and AUC values between 0.92 and 0.93. The narrow variation across folds confirms that the classifier learned consistent thermal–EDA patterns rather than memorizing the training data.

**Figure 5:**
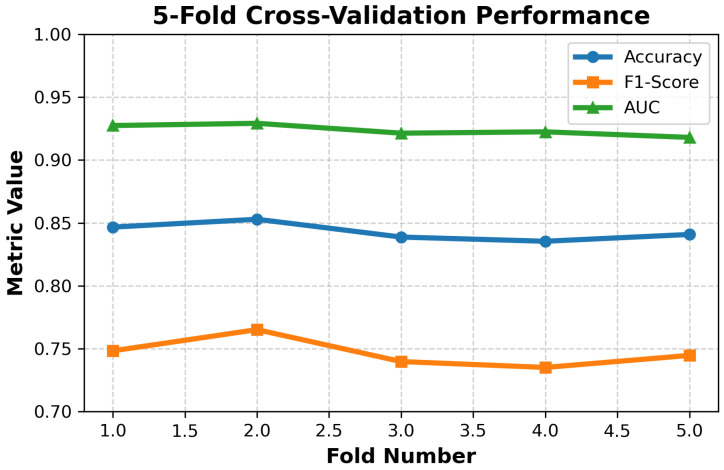
Five-fold cross-validation performance of the temperature-adjusted classification model, showing accuracy, F1-score, and AUC across folds. The narrow variance across folds indicates strong generalization and absence of overfitting.

The cross-validation accuracy (0.84–0.86) is slightly lower than the hold-out test accuracy (0.883). This difference reflects the stricter subject-independent partitioning enforced during cross-validation, which prevents data from the same individual from appearing in both training and validation folds. Consequently, the cross-validation results provide a more conservative and realistic estimate of generalization performance. The full cross-validation results are illustrated in Figure 5.

As shown in Table 9, feature importance analysis indicates that the temperature gradient and the conductance–temperature coupling ratio were among the most influential predictors. These were followed by the fused baseline deviation feature and short-term conductance descriptors.

These findings support the role of thermal dynamics as key determinants in differentiating thermoregulatory drift from genuine sympathetic activation. The combined feature set reflects both slow and rapid physiological adjustments, capturing the complex temporal structure of electrodermal responses.

Baseline statistical analysis further validated the separability of the thermal descriptors. Thermal drift, temperature gradient, the conductance–temperature ratio, and short-range conductance deviation each exhibited significant class-level differences in the analysis of variance tests. Mann–Whitney U comparisons between the Negative and Neutral classes confirmed statistically meaningful separations, particularly for the temperature gradient and the conductance–temperature ratio. These patterns align with the hypothesized thermal contribution to conductance variability and justify their inclusion in the final model.

The model correctly identified 1431 samples from the Other class and 688 samples from the Negative class, while producing 135 false positives and 146 false negatives. The pattern confirms that the classifier maintains balanced behavior for both classes despite the natural class imbalance. The relatively low number of cross-class errors demonstrates that the proposed temperature-adjusted framework effectively suppresses thermoregulatory noise and preserves stress-relevant electrodermal patterns, leading to stable discrimination between stress and non-stress segments across participants. Note that the classification report reflects only the 20% test partition (2400 samples: 1566 non-stress and 834 stress), whereas the earlier class distributions refer to the full 12,000-sample dataset.

Table 10 presents the complete classification report for the temperature-adjusted XGBoost model. The non-stress class achieved a precision of 0.907 and a recall of 0.914, indicating consistent detection stability. The stress class obtained a precision of 0.836 and a recall of 0.825, which is notable given the class imbalance and the rapid dynamics of stress-induced responses. The overall accuracy reached 0.883, with both macro and weighted F1-scores converging at approximately 0.88. These results demonstrate that the proposed temperature-adjusted framework maintains balanced predictive performance across classes while improving discriminability under varying thermal conditions.

The combined observations demonstrate that temperature-adjusted preprocessing, multiscale temporal modeling, and individualized normalization collectively produce a robust classification framework. The resulting AUC of 0.956 confirms that the proposed temperature-aware methodology successfully reconstructs stress-relevant electrodermal patterns while suppressing temperature-driven conductance fluctuations. This enhancement leads to more stable and interpretable stress assessment, with the temperature gradient and the conductance–temperature ratio emerging as the most influential predictors of class separability.

In addition, parameter sensitivity analysis was conducted for key model parameters, including learning rate and tree depth. The results show that the model remains stable across configurations, with performance variation below approximately 2%. A learning rate of 0.03 was selected to balance performance and generalization.

## 5. Discussion

The results confirm that temperature compensation improves the interpretability and stability of electrodermal signals under varying conditions. The application of the temperature-adjusted framework leads to clearer separation between thermal activity and stress-related conductance patterns, reduced temperature-driven drift, and improved feature stability across the dataset. These observations indicate that unadjusted electrodermal signals conflate sympathetic activation with thermoregulatory processes, obscuring physiologically meaningful stress dynamics.

Statistical analyses and feature behavior confirm that thermoregulatory influences introduce systematic conductance variations, particularly under elevated or localized thermal conditions common in immersive VR settings. Peripheral temperature changes affected conductance trends, revealing the limitations of earlier VR stress detection approaches that rely on fixed thresholds and overlook gradual thermal drift. Explicit thermal modeling mitigates these effects and restores structure aligned with affective arousal.

Feature-level behavior further supports this interpretation. Temperature-adjusted conductance measures exhibited improved stability across labeled segments, while temperature-derived descriptors such as short-term gradients and transition rates captured gradual thermal behavior without overwhelming conductance dynamics. The resulting fused feature representation produced more consistent patterns across labeled segments, thereby improving robustness under varying thermal conditions.

Adaptive baseline deviation mapping played a central role in stabilizing the adjusted signals. The adaptive scaling mechanism compensated for nonlinear and subject-specific temperature–EDA relationships, while proportional adjustment removed predictable thermal components. Together, these mechanisms strengthened alignment between conductance changes and stress-related activity, enabling clearer differentiation between emotional and thermoregulatory sweating.

Thermal descriptors themselves contributed meaningful discriminatory power. The temperature gradient (TempGradient) showed clear separation across affective classes (ANOVA F=852.99, $p<10^{-8}$), while the conductance–temperature coupling ratio (CouplingRatio) provided the strongest distinction ($F=1.11\times 10^{4}$, $p<10^{-8}$). These findings indicate that short-term thermal dynamics and temperature–EDA interactions contain stable, stress-relevant information when appropriately contextualized.

Importantly, the proposed framework remains computationally lightweight. Both proportional and adaptive compensation operate with constant-time updates, making the approach suitable for real-time deployment on standalone VR headsets and edge hardware. This aligns with the practical objective of minimizing hardware complexity while maintaining reliable stress monitoring in latency-sensitive immersive applications.

A limitation of this study is the reliance on laboratory data rather than fully interactive VR recordings. The WESAD dataset does not capture motion artifacts, device-induced heating, or environmental variability typical of real VR use. Future work should therefore evaluate the framework within fully integrated VR systems under realistic interaction and thermal conditions. Future work will extend this framework to fully immersive VR environments to evaluate the combined effects of motion, interaction dynamics, and device-induced thermal conditions on stress detection performance.

While the current validation is conducted using a controlled laboratory dataset WESAD, the proposed framework is designed for deployment in immersive VR environments where additional thermal and motion-related factors are present. Future work will include direct experimental validation under VR conditions, incorporating HMD-induced heating, user motion, and extended immersion effects to further evaluate real-world applicability.

The combined normalization strategy was selected to improve subject-independent robustness by capturing both long-term physiological baselines and short-term session-specific variations. This design is particularly important for deployment in dynamic environments such as VR, where both inter-subject variability and environmental fluctuations can significantly affect physiological signals. In practical VR deployment, additional factors such as user motion, sensor noise, and hardware-induced thermal variation may affect performance. In particular, motion artifacts and HMD heating can introduce variability not present in controlled datasets such as WESAD. As a result, a moderate reduction in classification accuracy is expected under real-world VR conditions. However, the proposed temperature-adjusted framework mitigates these effects by explicitly modeling thermal dynamics, improving robustness compared to conventional EDA-based methods. From a computational perspective, the TASD framework is lightweight and suitable for real-time implementation. The proportional adjustment and adaptive scaling operations require only simple arithmetic computations per time step, resulting in linear time complexity O(N) with minimal memory overhead. The classification stage, based on gradient boosting, can be efficiently executed on standard CPUs and embedded processors commonly available in VR systems. This makes the framework compatible with standalone VR headsets and edge devices without requiring specialized hardware.

This study is based on dataset-driven analysis using the WESAD dataset under controlled laboratory conditions. Clinical or real-world validation was beyond the scope of the current study. The present study focuses on methodological evaluation within a structured setting. The results demonstrate clear improvements in modeling temperature–physiological interactions. Further experimental studies in real-world and immersive VR environments will extend the evaluation by examining system behavior under dynamic conditions and enabling quantitative ground truth analysis.

In summary, integrating temperature adjustment into electrodermal stress modeling improves signal interpretability, feature stability, and stress specificity. Also, these results further confirm that the proposed normalization strategy and parameter selection contribute to stable and robust subject-independent performance. By separating sympathetic activity from thermoregulatory influences, the proposed framework provides a physiologically grounded and practically deployable basis for reliable stress monitoring in VR/XR environments.

## 6. Conclusions

This work introduces a temperature-adjusted framework for separating stress-induced electrodermal activity from thermoregulatory conductance changes in VR/XR settings. By combining proportional and adaptive thermal compensation with deviation-based normalization, the approach enables EDA to be interpreted more reliably under changing thermal conditions. The validation analysis shows that thermal modeling improves the physiological specificity of conductance features and strengthens separation between stress and non-stress segments.

The adaptive scaling model provides clearer condition-dependent behavior than the proportional method, and temperature–EDA features remain stable even when peripheral temperature increases. Participant-independent evaluation confirms that the framework generalizes across individuals. These results further confirm that temperature-aware signal processing is essential for accurate stress detection in immersive environments.

The innovation of this work lies in treating temperature and conductance as coupled processes rather than independent channels, and in providing a lightweight adjustment mechanism suitable for real-time VR deployment. By reducing temperature-driven drift and aligning conductance changes with affective events, the framework yields signals that are more stable, interpretable, and usable in practical VR/XR systems. The conclusions of this study are based on dataset-driven analysis, providing a controlled setting for evaluating the proposed framework. While additional experimental validation is needed to further assess real-world performance, the current results demonstrate consistent and statistically supported improvements in temperature-adjusted electrodermal modeling. In particular, future studies will quantitatively evaluate the effectiveness of the proposed temperature-compensation method in distinguishing environmental temperature variations from emotion-induced physiological responses.

## 7. Data Availability Statement

The dataset used in this study The WESAD dataset used in this study is publicly available online. Preprocessing, feature extraction, and model configuration details are provided in the manuscript to ensure reproducibility.

## Figures and Tables

**Table 1:** Thermal subset from WESAD used in this study.

Aspect	Items	Notes
Signals	EDA, Temperature	EDA low-pass 1 Hz; temperature EMA smoothing
Baselines	Global, Individual, Pre-task	Deviations in Equations (4)–(6)
Temperature terms	TEMP_Literature, TEMP_Simulated	Thermal predictors
Labels	1 Neutral, 2 Negative, 3 Positive	Binary: 2 → Stress; 1 + 3 → No-Stress
Scale	12,000 rows; imbalanced (4000 vs. 8000)	Participant-stratified folds

**Table 2:** Feature groups used after thermal adjustment.

Group	Examples	Purpose
EDA (adjusted)	Mean, SCR count, amplitude, rise time	Sympathetic activation
Temperature	Gradient, local change, EMA	Thermal context
Deviation	Δ${\mathrm{EDA}}_{\mathrm{global}}$, Δ${\mathrm{EDA}}_{\mathrm{indiv}}$, Δ${\mathrm{EDA}}_{\mathrm{pre}}$	Baseline normalization
Fusion	Concatenated vectors	Joint inference

**Table 3:** Thermal TASD parameter definitions and default values.

Parameter	Description	Default Value
$f_{c}^{\mathrm{EDA}}$	EDA low-pass cutoff	1.0 Hz
$\tau_{\mathrm{EMA}}$	Temperature EMA window	5 s
$\lambda_{\mathrm{prop}}$	Proportional coefficient	session-fit
$k_{\mathrm{adapt}}$	Adaptive update rate	$10^{-3}$
*L*	Coupling window length	30 s
$\epsilon_{\Delta }$	Numerical tolerance	$10^{-4}$
$w_{g},w_{i},w_{p}$	Baseline weights	correlation-driven
$\theta_{\mathrm{SCR}}$	SCR threshold	0.05 µS
$\beta_{\mathrm{reg}}$	Ridge regularization	$10^{-2}$
$\alpha_{\mathrm{pre}}$	Pre-task fraction	0.20

**Table 4:** Statistical summary of thermal features across the three affective classes and the binary Stress–No-Stress mapping. Values include class means, standard deviations, ANOVA F-statistics, and Mann–Whitney U-test results.

Feature	Class	Mean	SD	N	ANOVA F (*p*)	U-Test U (*p*)
TempGradient	Negative	0.1446	0.6858	4000	852.99 (<$10^{-8}$)	1.84 × $10^{7}$ (<$10^{-8}$)
	Neutral	−0.3848	0.8103	4000		
	Positive	0.2287	0.6550	4000		
CouplingRatio	Negative	0.1685	0.0348	4000	1.11 × $10^{4}$ (<$10^{-8}$)	7.94 × $10^{6}$ (<$10^{-8}$)
	Neutral	0.2480	0.0231	4000		
	Positive	0.1726	0.0204	4000		
ThermalDrift	Negative	34.5678	0.3324	4000	3.30 (0.0368)	1.64 × $10^{7}$ (0.0324)
	Neutral	34.5567	0.3271	4000		
	Positive	34.5491	0.3215	4000		

**Table 5:** Ablation study of temperature adjustment components under subject-independent evaluation.

Configuration	Accuracy	AUC
RAW (Unadjusted EDA)	0.8696	0.9441
PA-only	0.8546	0.9341
AS-only	0.8646	0.9417
TASD (PA + AS)	0.8529	0.9374

**Table 6:** Impact of baseline normalization strategies on classification performance.

Normalization Strategy	Accuracy	Notes
Global Baseline Only	0.792	Lacks subject adaptation
Individual Baseline Only	0.801	Limited session awareness
Combined (TASD)	0.853	Full normalization (global + individual + pre-task)

**Table 7:** Performance of the temperature-adjusted extreme gradient boosting (XGBoost) classifier.

Metric	Value
Accuracy	0.883
F1-score	0.815
AUC	0.956

**Table 8:** Comparison of classification models using TASD feature set under subject-independent evaluation.

Model	Accuracy	AUC
Logistic Regression	0.902	0.952
SVM (RBF)	0.905	0.966
Random Forest	0.898	0.960
Gradient Boosting	0.901	0.958
XGBoost (TASD)	0.883	0.956

**Table 9:** Feature importance ranking for the temperature-adjusted XGBoost model.

Feature	Importance
Temperature gradient	0.390
Conductance–temperature ratio	0.325
Fused baseline deviation	0.167
Short-term conductance change	0.067
Thermal drift	0.035
Temperature acceleration	0.017

**Table 10:** Classification report for the temperature-adjusted XGBoost model. Values include per-class precision, recall, F1-score, and support, along with overall and averaged metrics.

Class	Precision	Recall	F1-Score	Support
0 (Non-stress)	0.907	0.914	0.911	1566
1 (Stress)	0.836	0.825	0.830	834
Accuracy	0.883	0.883	0.883	2400
Macro avg	0.872	0.869	0.871	2400
Weighted avg	0.883	0.883	0.883	2400

## Data Availability

Publicly available datasets were analyzed in this study.

## References

[1] Sano A., Taylor S., McHill A.W., Phillips A.J., Barger L.K., Klerman E., Picard R. (2018). Identifying objective physiological markers and modifiable behaviors for self-reported stress and mental health status using wearable sensors and mobile phones: Observational study. J. Med. Internet Res..

[2] Posada-Quintero H.F., Chon K.H. (2020). Innovations in electrodermal activity data collection and signal processing: A systematic review. Sensors.

[3] Anderson A.P., Mayer M.D., Fellows A.M., Cowan D.R., Hegel M.T., Buckey J.C. (2017). Relaxation with immersive natural scenes presented using virtual reality. Aerosp. Med. Hum. Perform..

[4] Kreibig S.D. (2010). Autonomic nervous system activity in emotion: A review. Biol. Psychol..

[5] Boucsein W. (2012). Electrodermal Activity.

[6] Hernandez J., Morris R.R., Picard R.W. Call Center Stress Recognition with Person-Specific Models. Proceedings of the 2014 International Conference on Multimodal Interaction (ICMI 2014).

[7] Bach D.R., Friston K.J., Dolan R.J. (2010). Analytic measures for quantification of arousal from spontaneous skin conductance fluctuations. Int. J. Psychophysiol..

[8] Posada-Quintero H.F., Florian J.P., Orjuela-Cañón A.D., Aljama-Corrales T., Charleston-Villalobos S., Chon K.H. (2016). Power spectral density analysis of electrodermal activity for sympathetic function assessment. Ann. Biomed. Eng..

[9] Eggert L.D., Kleinstäuber M., Hiller W., Witthöft M. (2017). Emotional interference and attentional processing in premenstrual syndrome. J. Behav. Ther. Exp. Psychiatry.

[10] Seto G., Landry M., Amft O. Modeling the Influence of Temperature on Electrodermal Activity. Proceedings of the 2019 IEEE International Conference on Pervasive Computing and Communications Workshops (PerCom Workshops).

[11] Rah A., Chen Y. (2025). Virtual Reality as a Stress Measurement Platform: Real-Time Behavioral Analysis with Minimal Hardware. Sensors.

[12] Smets E., Casale P., Großekathöfer U., Lamichhane B., De Raedt W., Bogaerts K., Van Diest I., Van Hoof C. (2018). Comparison of machine learning techniques for psychophysiological stress detection. Pervasive Computing Paradigms for Mental Health.

[13] Asahina M., Suzuki A., Mori M., Kanesaka T., Hattori T. (2003). Emotional sweating response in a patient with bilateral amygdala damage. Int. J. Psychophysiol..

[14] Iwase S., Ikeda T., Kitazawa H., Hakusui S., Sugenoya J., Mano T. (1997). Altered response in cutaneous sympathetic outflow to mental and thermal stimuli in primary palmoplantar hyperhidrosis. J. Auton. Nerv. Syst..

[15] Cheung S.S. (2010). Interconnections between thermal perception and exercise capacity in the heat. Scand. J. Med. Sci. Sport..

[16] Kenny G.P., Journeay W.S. (2010). Human thermoregulation: Separating thermal and nonthermal effects on heat loss. Front. Biosci..

[17] Kuno Y. (1956). Human Perspiration.

[18] Vetrugno R., Liguori R., Cortelli P., Montagna P. (2003). Sympathetic skin response: Basic mechanisms and clinical applications. Clin. Auton. Res..

[19] Jamali A., Alipour S., Rah A. (2024). Leveraging Weak Supervision and BiGRU Neural Networks for Sentiment Analysis on Label-Free News Headlines. Proceedings of the 2024 IEEE 3rd International Conference on AI in Cybersecurity (ICAIC).

[20] Molokwu B.C., Rah A., Molokwu R.C. (2025). Fine-Grained Sentiment Mining at Document Level on Big Data Using a State-of-the-Art Representation-Based Transformer (ModernBERT). Book Chapter. https://www.researchgate.net/.

[21] Alipour S., Rah A., Karki B., Burris J., Coward L., Liao T. Enhancing Physics Education: Designing Customized Virtual Reality for Teaching Crystalline Structures. Proceedings of the 2024 IEEE International Symposium on Mixed and Augmented Reality Adjunct (ISMAR-Adjunct).

[22] Shiban Y., Diemer J., Müller J., Brütting-Schick J., Pauli P., Mühlberger A. (2016). Diaphragmatic breathing during virtual reality exposure therapy for aviophobia: Functional coping strategy or avoidance behavior? A pilot study. BMC Psychiatry.

[23] Gaggioli A., Pallavicini F., Morganti L., Serino S., Scaratti C., Briguglio M., Crifaci G., Vetrano N., Giulintano A., Bernava G. (2014). Experiential virtual scenarios with real-time monitoring (interreality) for the management of psychological stress: A block randomized controlled trial. J. Med. Internet Res..

[24] Rah A., Chen Y. (2025). Virtual Reality Centric Stress Detection Using Dynamic Baseline Calibration. Electronics.

[25] Schmidt P., Reiss A., Dürichen R., Marberger C., Van Laerhoven K. (2018). Introducing WESAD, a multimodal dataset for wearable stress and affect detection. arXiv.

[26] Chen T., Guestrin C. XGBoost: A scalable tree boosting system. Proceedings of the 22nd ACM SIGKDD International Conference on Knowledge Discovery and Data Mining.

